# BIX02189 Suppresses Adipogenesis and Lipid Accumulation Through Inhibition of MEK5-STAT3/STAT5 Signaling and Activation of AMPK in Adipocytes and Zebrafish

**DOI:** 10.3390/ijms27146468

**Published:** 2026-07-21

**Authors:** Nivethasri Lakshmana Perumal, Muneer Hussain, Dae-Gu Son, Jacqueline M. Stephens, Gi-Young Park, Byeong-Churl Jang

**Affiliations:** 1 Department of Molecular Medicine, College of Medicine, Keimyung University, 1095 Dalgubeoldaero, Dalseo-gu, Daegu 42601, Republic of Korea; nivi1998@naver.com (N.L.P.); muneerhussain39@naver.com (M.H.); 2 Department of Plastic Surgery, College of Medicine, Keimyung University, Daegu 42601, Republic of Korea; handson@dsmc.or.kr; 3 Adipocyte Biology Laboratory, Pennington Biomedical Research Center, Louisiana State University, Baton Rouge, LA 70808, USA; jsteph1@lsu.edu; 4 Department of Rehabilitation Medicine, Daegu Catholic University School of Medicine, Nam-Gu, Daegu 42472, Republic of Korea

**Keywords:** BIX02189, MEK5, adipogenesis, obesity, AMPK, zebrafish

## Abstract

Obesity is a major metabolic disorder characterized by excessive lipid accumulation and adipocyte differentiation. The mitogen-activated protein kinase kinase 5 (MEK5) signaling pathway has been implicated in diverse cellular processes; however, its role in adipogenesis remains incompletely understood. In this study, we investigated the anti-adipogenic effects of BIX02189, a selective MEK5 inhibitor, using 3T3-L1 adipocytes, human adipose-derived stem cells (hASCs), and zebrafish models. Treatment with BIX02189 significantly reduced lipid accumulation and triglyceride content during adipocyte differentiation in a dose-dependent manner without marked cytotoxicity. BIX02189 effectively suppressed MEK5 phosphorylation and downregulated the expression of key adipogenic transcription factors, including peroxisome proliferator-activated receptor gamma (PPAR-γ) and CCAAT/enhancer-binding protein alpha (C/EBP-α). In addition, BIX02189 decreased the phosphorylation of signal transducer and activator of transcription 3 (STAT3) and STAT5, as well as the expression of lipogenic markers such as fatty acid synthase (FAS), perilipin A, and leptin. Conversely, BIX02189 enhanced AMP-activated protein kinase (AMPK) phosphorylation and markedly reduced the protein and mRNA expression of acetyl-CoA carboxylase (ACC), a key enzyme involved in fatty acid synthesis. Similar anti-adipogenic effects were observed in hASCs. Furthermore, BIX02189 significantly attenuated lipid accumulation in a zebrafish obesity model without affecting body length or causing overt toxicity. Collectively, these findings demonstrate that pharmacological inhibition of MEK5 suppresses adipogenesis and lipid accumulation through regulation of the STAT3/STAT5–PPAR-γ axis and activation of AMPK signaling. These findings provide the first evidence that MEK5 inhibition exerts anti-adipogenic effects in adipocytes and zebrafish, highlighting the MEK5 signaling pathway as a previously unrecognized regulator of adipogenesis and lipid metabolism.

## 1. Introduction

Obesity is a major global health challenge and a significant risk factor for numerous metabolic disorders, including hyperlipidemia, type 2 diabetes mellitus, cardiovascular disease, non-alcoholic fatty liver disease, and certain cancers [1]. The global prevalence of obesity has increased dramatically over the past several decades, placing a substantial burden on healthcare systems and socioeconomic development. According to the NCD Risk Factor Collaboration (NCD-RisC), the prevalence of adult obesity more than doubled between 1990 and 2022, with over one billion adults living with obesity worldwide in 2022. These alarming statistics underscore obesity as one of the most urgent public health challenges requiring the development of effective preventive and therapeutic strategies [2]. Excess caloric intake and reduced energy expenditure promote adipocyte hypertrophy and hyperplasia, resulting in excessive lipid accumulation, primarily in the form of triglycerides, and ultimately contributing to the development and progression of obesity [3,4].

Adipogenesis is a highly coordinated biological process through which fibroblast-like preadipocytes differentiate into mature adipocytes capable of storing large amounts of lipids [5]. This process is tightly regulated by a complex transcriptional network involving CCAAT/enhancer-binding proteins (C/EBPs) and peroxisome proliferator-activated receptor gamma (PPAR-γ), which function as master regulators of adipocyte differentiation [6,7]. In addition, signaling pathways such as Janus kinase (JAK)/signal transducer and activator of transcription (STAT) contribute significantly to adipocyte differentiation, lipid metabolism, and adipose tissue homeostasis [8]. Lipogenic enzymes, including fatty acid synthase (FAS) and acetyl-CoA carboxylase (ACC), together with lipid droplet-associated proteins such as perilipin A, play essential roles in fatty acid synthesis, lipid storage, and adipocyte maturation [9,10,11,12]. Furthermore, metabolic regulators including AMP-activated protein kinase (AMPK), protein kinase A (PKA), extracellular signal-regulated kinase (ERK), and cyclic AMP (cAMP) coordinate adipogenesis and energy metabolism by integrating nutritional and hormonal signals [10,13].

AMPK functions as a central metabolic sensor that maintains cellular energy homeostasis by suppressing anabolic pathways and stimulating catabolic processes [14]. Activation of AMPK inhibits lipogenesis through the regulation of downstream targets, including acetyl-CoA carboxylase (ACC), thereby reducing fatty acid synthesis and lipid accumulation [15]. Although liver kinase B1 (LKB1) is widely recognized as a major upstream kinase of AMPK, accumulating evidence suggests that AMPK can also be activated through LKB1-independent mechanisms under specific physiological and pathological conditions [16]. However, the relationship between MEK5 signaling and AMPK-mediated metabolic regulation during adipogenesis remains poorly understood.

Among the mitogen-activated protein kinase (MAPK) family members, the MEK5–ERK5 signaling pathway has been implicated in the regulation of cellular proliferation, differentiation, survival, stress responses, and metabolic homeostasis [17]. Emerging evidence indicates that MEK5 signaling is involved in the regulation of cellular differentiation and metabolic homeostasis; however, its role in adipogenesis remains poorly understood. Because STAT3 and STAT5 are well-established regulators of adipocyte differentiation, it is plausible that MEK5 signaling interacts with STAT-dependent transcriptional pathways to regulate adipogenesis. However, this potential relationship has not yet been systematically investigated. Nevertheless, the precise role of MEK5 signaling in adipocyte differentiation and obesity-associated lipid accumulation remains largely unknown. In particular, whether MEK5 regulates adipogenesis through coordinated modulation of STAT signaling and metabolic pathways such as AMPK has not yet been elucidated.

BIX02189 is a selective pharmacological inhibitor of MEK5 that has been extensively used as a molecular tool to investigate MEK5–ERK5 signaling in cancer biology, inflammation, and cellular stress responses [18,19,20]. The zebrafish model has emerged as a versatile tool due to their physiological and genetic similarities to mammals; it represents a practical model for studying lipid metabolism, obesity pathogenesis, and the capabilities of anti-adipogenic compounds [21]. Despite its widespread use, little is known regarding the potential role of BIX02189 in adipogenesis and obesity-related metabolic regulation. Given the central importance of intracellular signaling networks in controlling adipocyte differentiation and lipid metabolism, targeting MEK5 signaling may represent a novel therapeutic strategy for obesity and related metabolic disorders.

Therefore, in the present study, we investigated the anti-adipogenic effects of BIX02189 using both in vitro and in vivo experimental models. Murine 3T3-L1 preadipocytes are among the most extensively characterized in vitro models for investigating adipocyte differentiation, lipid metabolism, and the molecular mechanisms underlying adipogenesis [22]. Human adipose-derived stem cells (hASCs) provide a physiologically and clinically relevant human model for studying adipocyte differentiation and evaluating compounds that modulate adipogenic and lipogenic signaling pathways. In addition, zebrafish have emerged as a valuable vertebrate model for obesity research because diet-induced obesity in zebrafish closely recapitulates key pathological features of mammalian obesity, including excessive lipid accumulation and increased adiposity, thereby providing an effective in vivo platform for evaluating compounds that regulate lipid metabolism and obesity-associated phenotypes [23,24]. We examined the effects of BIX02189 on adipocyte differentiation and lipid accumulation in murine 3T3-L1 preadipocytes and human adipose-derived stem cells (hASCs) and further evaluated its efficacy in a zebrafish model of diet-induced obesity. In contrast, we explored the underlying molecular mechanisms focusing on the MEK5–STAT3/STAT5 signaling axis and AMPK-mediated metabolic regulation. Our findings provide evidence that pharmacological inhibition of MEK5 suppresses adipogenesis and lipid accumulation through modulation of adipogenic transcription factors and activation of an apparent LKB1-independent AMPK signaling pathway, identifying the MEK5 signaling pathway as a novel mediator of adipocyte differentiation and lipid accumulation that warrants further investigation.

## 2. Results

### 2.1. Screening of Protein Kinase Inhibitors Identifies BIX02189 as a Potent Anti-Adipogenic Candidate

**Figure 1:**
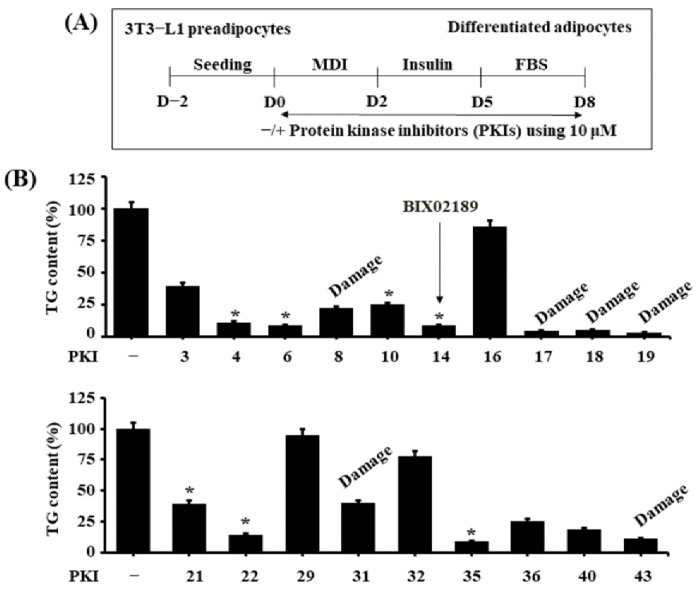
Screening of protein kinase inhibitors identifies BIX02189 as a potent inhibitor of adipogenesis in 3T3-L1 cells. (**A**) Schematic illustration of the screening protocol. 3T3-L1 preadipocytes were induced to differentiate using a standard adipogenic differentiation protocol and treated with individual protein kinase inhibitors (PKIs; 10 μM) throughout the differentiation period. Intracellular triglyceride (TG) content was measured on day 8 (D8) as an indicator of adipocyte differentiation and lipid accumulation. (**B**) Quantitative analysis of intracellular TG content in differentiated 3T3-L1 adipocytes following treatment with various PKIs. Among the compounds tested, BIX02189 exhibited the strongest inhibitory effect on TG accumulation compared with differentiated control cells. Data are presented as the percentage of TG content relative to the differentiated control group. Data are presented as the mean ± SE from at least three independent experiments. * *p* < 0.05 versus the control group. Several PKIs (Nos. 8, 17, 18, 19, 31, and 43) exhibited marked cytotoxicity (Damage) under the screening conditions.

Figure 1A illustrates the experimental screening strategy used to identify novel anti-adipogenic compounds from a library of protein kinase inhibitors (PKIs). Briefly, 3T3-L1 preadipocytes were induced to differentiate under standard adipogenic conditions and treated with individual PKIs (10 μM) throughout the differentiation period. Intracellular triglyceride (TG) content was measured at the end of differentiation as a quantitative indicator of adipogenesis. Among the tested PKIs, several compounds moderately reduced intracellular TG accumulation; however, BIX02189 exhibited the most pronounced inhibitory effect on lipid accumulation (Figure 1B). Treatment with BIX02189 resulted in a substantial reduction in TG content compared with differentiated control cells, indicating potent suppression of adipocyte differentiation. Because BIX02189 targets MEK5 signaling, these findings suggest a previously unrecognized role for the MEK5 pathway in adipogenesis and lipid metabolism. Based on its superior anti-adipogenic activity, BIX02189 was selected as the lead compound for subsequent validation and mechanistic studies aimed at elucidating its effects on adipogenic signaling pathways and metabolic regulation.

### 2.2. BIX02189 Dose-Dependently Suppresses Adipocyte Differentiation and Lipid Accumulation in 3T3-L1 Cells

**Figure 2:**
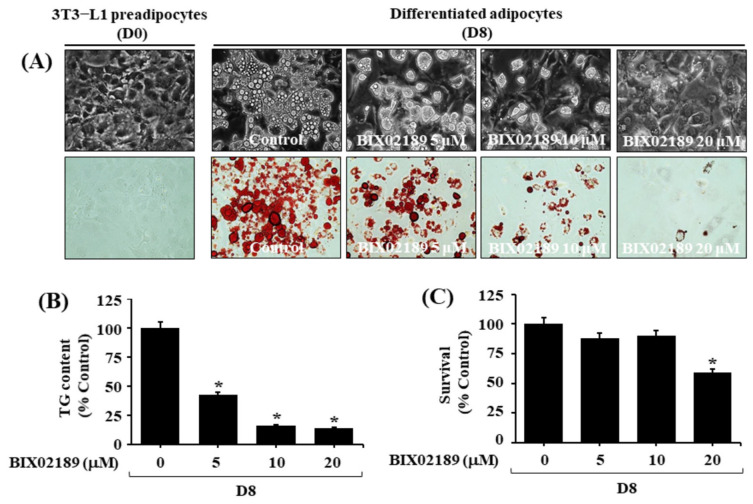
BIX02189 suppresses adipocyte differentiation and lipid accumulation in 3T3-L1 cells in a dose-dependent manner. (**A**) Representative phase-contrast and Oil Red O-stained images of 3T3-L1 cells. Preadipocytes (D0) were induced to differentiate and treated with BIX02189 (0, 5, 10, and 20 μM) throughout the differentiation period (D0–D8). Control cells exhibited extensive lipid droplet formation, whereas BIX02189 treatment reduced lipid accumulation in a concentration-dependent manner. (**B**) Quantitative analysis of intracellular triglyceride (TG) content in differentiated 3T3-L1 adipocytes. TG levels were significantly decreased following BIX02189 treatment in a dose-dependent manner. (**C**) Cell viability of 3T3-L1 cells treated with BIX02189 for 8 days. Cell survival was expressed as a percentage of the control group. BIX02189 at 5 and 10 μM had minimal effects on cell viability, whereas a moderate reduction was observed at 20 μM. Data are presented as mean ± SE from at least three independent experiments. * *p* < 0.05 versus control.

Following the identification of BIX02189 as a potent anti-adipogenic candidate, its effects on adipocyte differentiation were further evaluated in 3T3-L1 cells. As shown in Figure 2A, untreated control cells underwent robust adipogenic differentiation and accumulated numerous large lipid droplets by day 8 (D8). In contrast, treatment with BIX02189 markedly reduced lipid droplet formation in a concentration-dependent manner. While 5 μM BIX02189 moderately decreased lipid accumulation, treatment with 10 μM resulted in a substantial reduction in both the size and number of lipid droplets. Notably, lipid accumulation was almost completely abolished in cells treated with 20 μM BIX02189, as demonstrated by phase-contrast microscopy and Oil Red O staining. Consistent with these morphological observations, quantitative analysis revealed a significant dose-dependent reduction in intracellular triglyceride (TG) content following BIX02189 treatment (Figure 2B). TG levels were significantly decreased at 5 μM and further reduced at 10 and 20 μM, indicating potent inhibition of adipocyte differentiation and lipid storage. To determine whether the reduction in lipid accumulation was associated with cytotoxicity, cell viability was assessed at the end of the differentiation period. As shown in Figure 2C, BIX02189 at 5 and 10 μM had minimal effects on cell viability, whereas a moderate reduction in cell survival was observed at 20 μM. These findings suggest that the inhibitory effects of BIX02189 on adipogenesis at 5–10 μM occur largely independently of cytotoxicity. Based on its potent anti-adipogenic activity and minimal effect on cell viability, 10 μM BIX02189 was selected for subsequent mechanistic studies.

### 2.3. BIX02189 Suppresses MEK5 Phosphorylation During 3T3-L1 Adipocyte Differentiation

**Figure 3:**
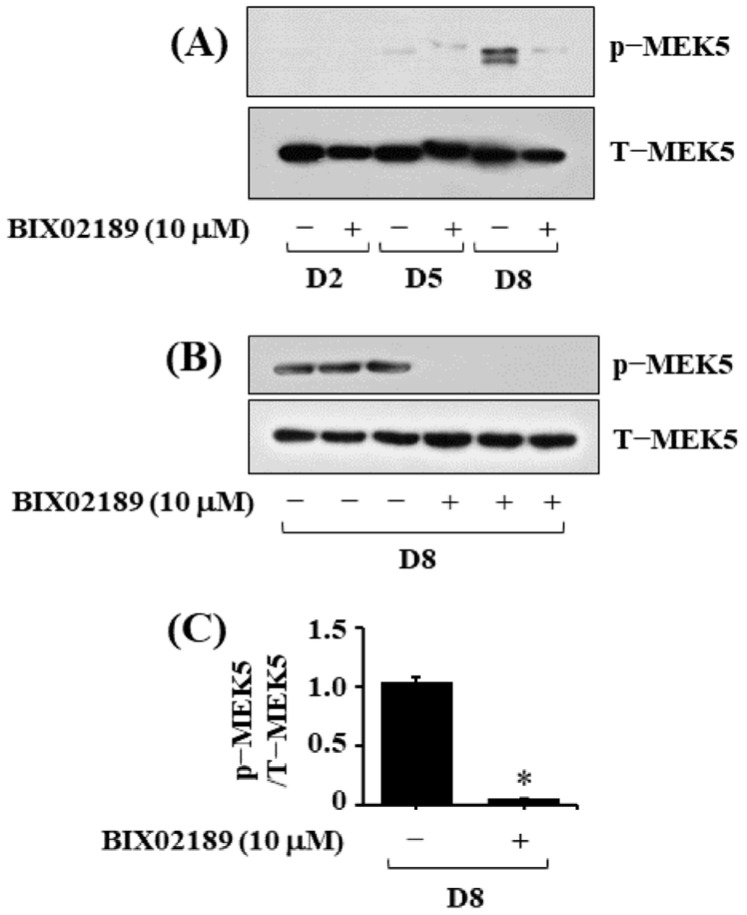
BIX02189 inhibits MEK5 phosphorylation during adipocyte differentiation in 3T3-L1 cells. (**A**) Representative Western blot analysis of phosphorylated MEK5 (p-MEK5) and total MEK5 (T-MEK5) during adipocyte differentiation. Cells were differentiated in the absence or presence of BIX02189 (10 μM), and protein samples were collected at D2, D5, and D8. BIX02189 markedly reduced MEK5 phosphorylation throughout the differentiation period without affecting total MEK5 expression. (**B**) Representative Western blot images from independent experiments performed at D8. BIX02189 consistently suppressed MEK5 phosphorylation, whereas total MEK5 protein levels remained largely unchanged. (**C**) Densitometric quantification of p-MEK5 normalized to total MEK5 at D8. Data are presented as mean ± SE from at least three independent experiments. * *p* < 0.05 versus control.

To verify whether BIX02189 effectively inhibits its molecular target during adipocyte differentiation, we examined the phosphorylation and expression levels of MEK5 in differentiating 3T3-L1 cells. As shown in Figure 3A, phosphorylation of MEK5 progressively increased during adipocyte differentiation, indicating activation of MEK5 signaling throughout the adipogenic process. In contrast, treatment with BIX02189 (10 μM) markedly reduced MEK5 phosphorylation at D2, D5, and D8 compared with the corresponding untreated controls. The full-length, uncropped immunoblots corresponding to Figure 3A are provided in Figure S1 respectively. Importantly, BIX02189 had little or no effect on total MEK5 protein expression, suggesting that the compound selectively inhibits MEK5 activation rather than MEK5 expression itself. To further validate this observation, independent immunoblot analyses were performed at D8. Consistent with the time-course results, BIX02189 reproducibly suppressed MEK5 phosphorylation without altering total MEK5 levels (Figure 3B). Additional representative images for Figure 3B are shown in Figure S2. Densitometric analysis of p-MEK5 normalized to total MEK5 confirmed a significant reduction in MEK5 activation following BIX02189 treatment (Figure 3C). These findings demonstrate that MEK5 signaling is activated during adipocyte differentiation and that BIX02189 effectively suppresses adipogenesis-associated MEK5 phosphorylation. Collectively, these results suggest that MEK5 activation contributes to adipocyte differentiation and reveal a previously uncharacterized role for MEK5 signaling in the regulation of adipocyte differentiation and lipid homeostasis.

### 2.4. BIX02189 Suppresses Adipogenic Transcription Factors Through Inhibition of STAT3/STAT5 Signaling During Adipocyte Differentiation

**Figure 4:**
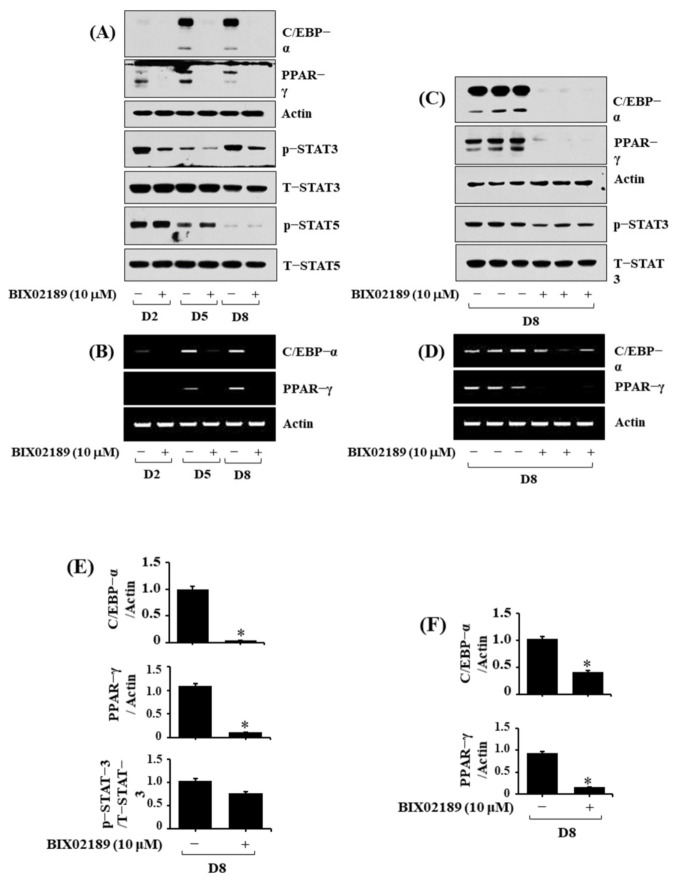
BIX02189 suppresses adipogenic transcription factors and STAT3/STAT5 signaling during adipocyte differentiation in 3T3-L1 cells. (**A**) Representative Western blot analysis of C/EBP-α, PPAR-γ, phosphorylated STAT3 (p-STAT3), total STAT3 (T-STAT3), phosphorylated STAT5 (p-STAT5), and total STAT5 (T-STAT5) in differentiating 3T3-L1 cells treated with or without BIX02189 (10 μM). Protein samples were collected at D2, D5, and D8. (**B**) RT-PCR analysis of C/EBP-α and PPAR-γ mRNA expression during adipocyte differentiation in the absence or presence of BIX02189 (10 μM). (**C**) Representative Western blot images from independent experiments performed at D8 showing the effects of BIX02189 on C/EBP-α, PPAR-γ, and STAT3 phosphorylation. (**D**) RT-PCR analysis from independent experiments performed at D8 confirms suppression of C/EBP-α and PPAR-γ mRNA expression by BIX02189. (**E**) Densitometric quantification of C/EBP-α, PPAR-γ and phosphorylated STAT3 normalized to β-actin and total STAT3 protein expression shown in (**C**). (**F**) Quantification of C/EBP-α and PPAR-γ mRNA expression normalized to total β-actin shown in (**D**). Data are presented as mean ± SE from at least three independent experiments. * *p* < 0.05 versus control.

To elucidate the molecular mechanisms underlying the anti-adipogenic activity of BIX02189, we investigated the expression of key adipogenic transcription factors and their upstream signaling molecules during 3T3-L1 adipocyte differentiation. As shown in Figure 4A, adipocyte differentiation was associated with progressive increases in the protein expression of C/EBP-α and PPAR-γ, accompanied by enhanced phosphorylation of STAT3 and STAT5 from D2 to D8 in untreated cells, indicating activation of adipogenic signaling pathways during differentiation. Treatment with BIX02189 (10 μM) markedly attenuated these differentiation-associated changes throughout the adipogenic process. Specifically, BIX02189 reduced the protein expression of both C/EBP-α and PPAR-γ at D2, D5, and D8. In parallel, phosphorylation of STAT3 and STAT5 was substantially decreased by BIX02189, whereas total STAT3 and STAT5 protein levels remained largely unchanged, suggesting selective inhibition of STAT activation rather than STAT expression. To confirm these observations, independent experiments were performed at D8. Consistent with the time-course analysis, BIX02189 markedly suppressed the protein expression of C/EBP-α and PPAR-γ and significantly reduced STAT3 phosphorylation (Figure 4C). Moreover, RT-PCR analysis demonstrated that BIX02189 significantly decreased the mRNA expression levels of both C/EBP-α and PPAR-γ (Figure 4B,D), indicating transcriptional repression of adipogenic regulators. Densitometric analysis further confirmed significant reductions in C/EBP-α and PPAR-γ protein expression as well as p-STAT3/STAT3 ratios following BIX02189 treatment (Figure 4E,F). Collectively, these findings indicate that BIX02189 suppresses adipocyte differentiation by inhibiting STAT3/STAT5 signaling and subsequently downregulating the master adipogenic transcription factors C/EBP-α and PPAR-γ.

### 2.5. BIX02189 Suppresses Lipogenic Gene Expression During Adipocyte Differentiation

**Figure 5:**
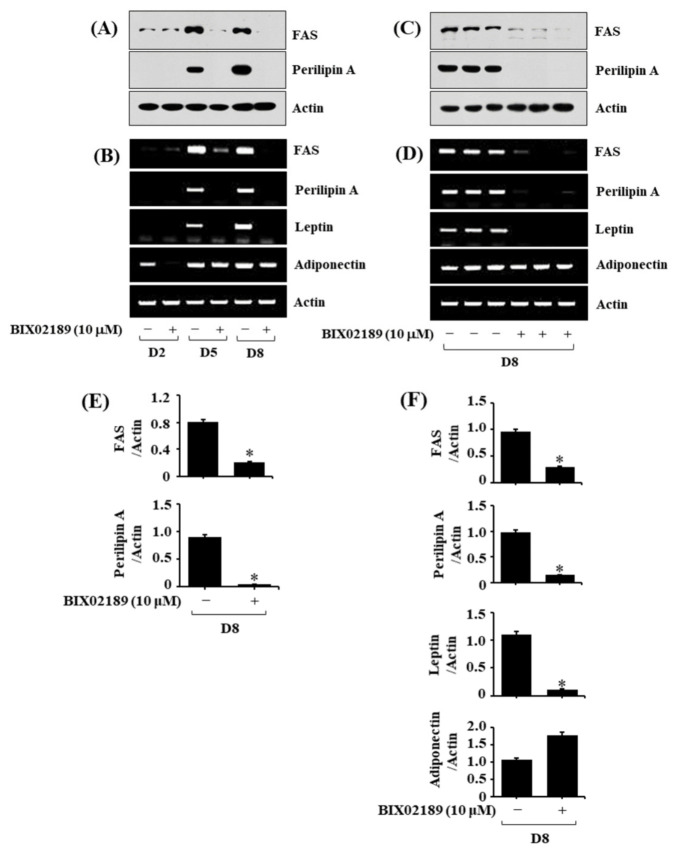
BIX02189 suppresses lipogenic gene expression during adipocyte differentiation in 3T3-L1 cells. (**A**) Representative Western blot analysis of fatty acid synthase (FAS) and perilipin A during adipocyte differentiation. Cells were differentiated in the absence or presence of BIX02189 (10 μM), and protein samples were collected at D2, D5, and D8. (**B**) RT-PCR analysis of FAS, perilipin A, leptin, and adiponectin mRNA expression during adipocyte differentiation in the absence or presence of BIX02189. (**C**) Representative Western blot images from independent experiments performed at D8 showing the effects of BIX02189 on FAS and perilipin A protein expression. (**D**) RT-PCR analysis from independent experiments performed at D8 confirms suppression of FAS, perilipin A, and leptin expression by BIX02189. (**E**) Densitometric quantification of FAS and perilipin A protein expression shown in (**C**). (**F**) Quantification of FAS, perilipin A, leptin, and adiponectin mRNA expression shown in (**D**). Data are presented as mean ± SE from at least three independent experiments. * *p* < 0.05 versus control.

To determine whether the anti-adipogenic effects of BIX02189 are associated with suppression of lipogenic pathways, we examined the expression of key lipogenic enzymes and adipocyte-associated markers during 3T3-L1 adipocyte differentiation. As shown in Figure 5A, the expression of fatty acid synthase (FAS) and perilipin A progressively increased during adipocyte differentiation in untreated control cells, reflecting enhanced lipogenesis and lipid droplet formation. Treatment with BIX02189 (10 μM) markedly attenuated the expression of both FAS and perilipin A throughout the differentiation period. The inhibitory effect was evident at D5 and became more pronounced at D8, suggesting sustained suppression of lipogenic signaling during adipocyte maturation. To further validate these findings, independent experiments were performed at D8. Consistent with the time-course analysis, BIX02189 significantly reduced the protein expression of FAS and perilipin A (Figure 5C). RT-PCR analysis further demonstrated that BIX02189 markedly decreased the mRNA expression of FAS, perilipin A, and leptin (Figure 5B,D). In contrast, adiponectin expression was relatively preserved compared with other adipogenic markers. Densitometric analysis confirmed significant reductions in FAS and perilipin A protein levels as well as leptin mRNA expression following BIX02189 treatment (Figure 5E,F). Collectively, these findings indicate that BIX02189 suppresses adipocyte differentiation not only by inhibiting adipogenic transcription factors but also by attenuating lipogenic gene expression and lipid storage programs. These results suggest that inhibition of MEK5 signaling suppresses downstream lipogenic pathways, thereby limiting lipid synthesis, lipid droplet formation, and adipocyte maturation.

### 2.6. BIX02189 Activates AMPK Independently of LKB1 and Suppresses ACC Expression During Adipocyte Differentiation

**Figure 6:**
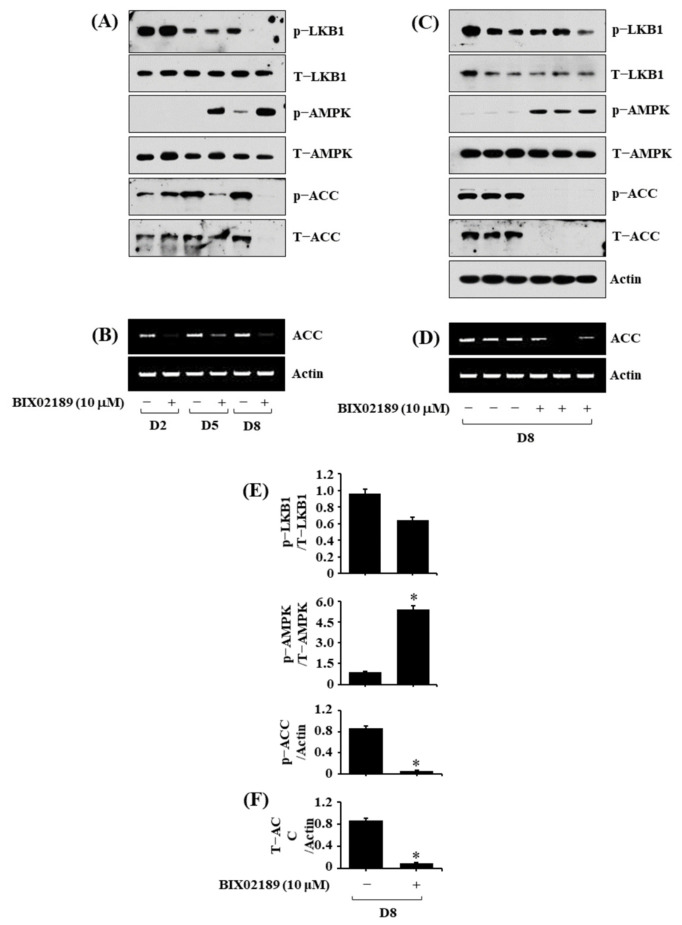
BIX02189 activates AMPK and suppresses ACC expression during adipocyte differentiation in 3T3-L1 cells. (**A**) Representative Western blot analysis of phosphorylated LKB1 (p-LKB1), total LKB1 (T-LKB1), phosphorylated AMPK (p-AMPK), total AMPK (T-AMPK), phosphorylated ACC (p-ACC), total ACC (T-ACC), and actin during adipocyte differentiation. Cells were differentiated in the absence or presence of BIX02189 (10 μM), and protein samples were collected at D2, D5, and D8. (**B**) RT-PCR analysis of ACC mRNA expression during adipocyte differentiation in the absence or presence of BIX02189. (**C**) Representative Western blot images from independent experiments performed at D8 showing the effects of BIX02189 on LKB1, AMPK, and ACC signaling. (**D**) RT-PCR analysis from independent experiments performed at D8 confirms suppression of ACC mRNA expression by BIX02189. (**E**) Densitometric quantification of p-LKB1/T-LKB1, p-AMPK/T-AMPK, p-ACC, and total ACC protein expression shown in (**C**). (**F**) Quantification of ACC mRNA expression shown in (**D**). Data are presented as mean ± SE from at least three independent experiments. * *p* < 0.05 versus control.

To further investigate the metabolic mechanisms underlying the anti-adipogenic effects of BIX02189, we examined the LKB1–AMPK signaling pathway and the expression of ACC, a key enzyme involved in fatty acid synthesis, during 3T3-L1 adipocyte differentiation. As shown in Figure 6A, phosphorylation of LKB1 progressively decreased following BIX02189 treatment throughout the differentiation period. In contrast, phosphorylation of AMPK was markedly increased, particularly at D5 and D8, despite the reduction in LKB1 phosphorylation. Total AMPK expression remained largely unchanged during the differentiation process. Interestingly, BIX02189 markedly reduced both phosphorylated ACC (p-ACC) and total ACC protein levels compared with untreated control cells. Consistent with the protein data, RT-PCR analysis revealed that ACC mRNA expression was significantly reduced following BIX02189 treatment (Figure 6B), indicating transcriptional suppression of ACC expression. To confirm these observations, independent experiments were performed at D8. As shown in Figure 6C, BIX02189 consistently decreased LKB1 phosphorylation while simultaneously enhancing AMPK phosphorylation. Moreover, both p-ACC and total ACC protein levels were markedly reduced. RT-PCR analysis further confirmed suppression of ACC mRNA expression (Figure 6D). Densitometric analysis demonstrated significant reductions in p-LKB1/T-LKB1, p-ACC, total ACC protein, and ACC mRNA expression, whereas AMPK phosphorylation was significantly increased following BIX02189 treatment (Figure 6E,F). Collectively, these findings suggest that BIX02189 activates AMPK through an apparent LKB1-independent mechanism and suppresses ACC expression at both the transcriptional and protein levels. This coordinated regulation may contribute to the inhibition of fatty acid synthesis and lipid accumulation during adipocyte differentiation. These results support a model in which MEK5 inhibition promotes LKB1-independent AMPK activation, leading to suppression of ACC-mediated lipogenesis and attenuation of adipocyte maturation.

### 2.7. BIX02189 Reduces Intracellular ATP Levels During Adipocyte Differentiation

**Figure 7:**
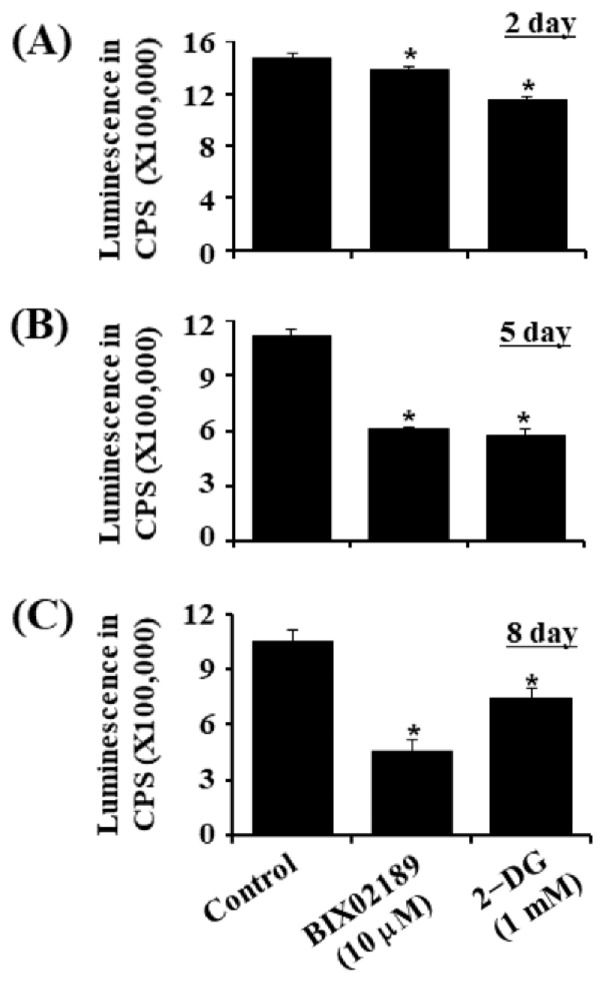
BIX02189 reduces intracellular ATP levels during adipocyte differentiation in 3T3-L1 cells. (**A**–**C**) Intracellular ATP levels were measured during adipocyte differentiation in the absence or presence of BIX02189 (10 μM). The glycolytic inhibitor 2-deoxy-D-glucose (2-DG) was included as a positive control for ATP depletion. ATP content was normalized to the corresponding control group. BIX02189 significantly reduced intracellular ATP levels throughout adipocyte differentiation, exhibiting a pattern comparable to that observed with 2-DG. Data are presented as mean ± SE from at least three independent experiments. * *p* < 0.05 versus control.

To further investigate the mechanism underlying AMPK activation following BIX02189 treatment, intracellular ATP levels were measured during adipocyte differentiation. Because 2-deoxy-D-glucose (2-DG), a well-established glycolytic inhibitor and ATP-depleting agent, induces cellular energy stress and activates AMPK, it was included as a positive control [25,26]. As shown in Figure 7A–C, untreated control cells maintained substantial intracellular ATP levels throughout adipocyte differentiation, consistent with the high metabolic demand associated with adipocyte maturation. In contrast, treatment with BIX02189 significantly reduced intracellular ATP levels throughout the differentiation period. The ATP-lowering effect of BIX02189 was evident at all examined time points and became more pronounced during the later stages of differentiation. Importantly, 2-DG also markedly reduced intracellular ATP levels, confirming the validity of the experimental system. Notably, the ATP-reducing effect of BIX02189 exhibited a pattern comparable to that observed with 2-DG, suggesting that BIX02189 induces a state of cellular energy stress. Given that AMPK functions as a central energy sensor activated in response to ATP depletion, the reduction in intracellular ATP levels may contribute to the enhanced AMPK phosphorylation observed following BIX02189 treatment. These findings provide a potential explanation for the activation of AMPK despite reduced LKB1 phosphorylation and support the possibility that BIX02189 activates AMPK through an alternative energy stress-associated mechanism. Collectively, these results suggest that BIX02189 suppresses adipocyte differentiation, at least in part, by reducing intracellular ATP levels and promoting AMPK activation, thereby contributing to inhibition of lipogenic pathways and adipocyte maturation.

### 2.8. BIX02189 Suppresses Adipogenic Differentiation in Human Adipose-Derived Stem Cells

**Figure 8:**
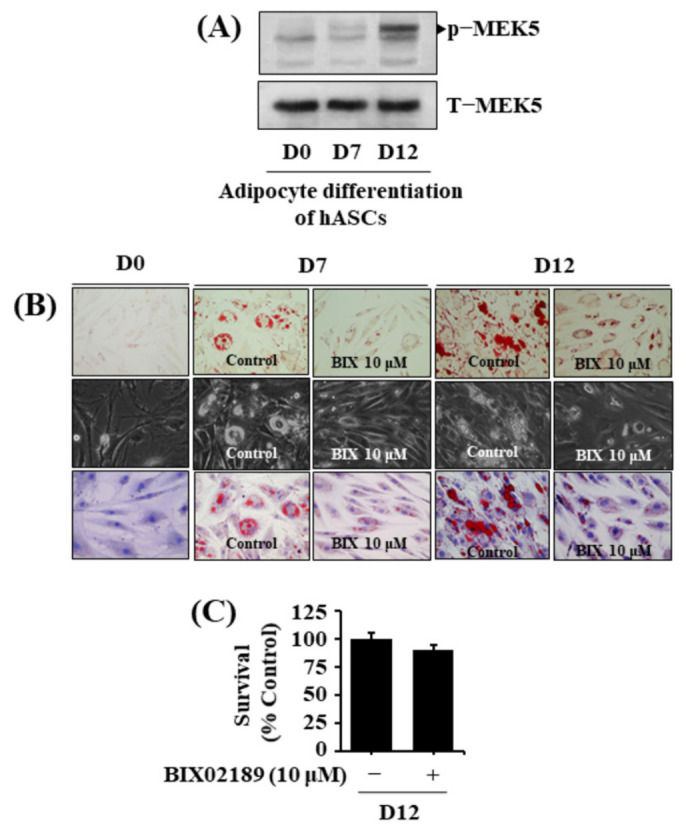
BIX02189 suppresses adipogenic differentiation and lipid accumulation in human adipose-derived stem cells (hASCs). (**A**) Representative Western blot analysis of phosphorylated MEK5 (p-MEK5) and total MEK5 (T-MEK5) during adipogenic differentiation of hASCs. Protein samples were collected at D0, D7, and D12. MEK5 phosphorylation progressively increased during adipocyte differentiation, whereas total MEK5 expression remained relatively unchanged. (**B**) Representative Oil Red O-stained images (**upper panels**), phase-contrast images (**middle panels**), and DAPI-stained images (**lower panels**) of hASCs differentiated in the absence or presence of BIX02189 (10 μM). Cells were cultured under adipogenic differentiation conditions and analyzed at D7 and D12. Control cells exhibited extensive lipid droplet accumulation, whereas BIX02189 markedly reduced intracellular lipid accumulation and adipocyte maturation. DAPI staining revealed comparable nuclear distribution between the different groups. (**C**) Cell viability of hASCs was expressed with and without BIX02189 (10 μM) at D12 as a percentage of control group. Data are presented as the mean ± SE from at least three independent experiments.

To determine whether the anti-adipogenic effects of BIX02189 observed in murine 3T3-L1 cells are reproducible in human cells, we evaluated MEK5 activation and adipogenic differentiation in human adipose-derived stem cells (hASCs). As shown in Figure 8A, adipogenic differentiation of hASCs was accompanied by a progressive increase in MEK5 phosphorylation from D0 to D12, whereas total MEK5 protein levels remained relatively unchanged. These findings indicate that activation of MEK5 signaling is associated with adipocyte differentiation not only in murine 3T3-L1 cells but also in human adipose-derived stem cells. To investigate the functional significance of MEK5 signaling in human adipogenesis, hASCs were differentiated in the absence or presence of BIX02189 (10 μM). Under adipogenic conditions, control cells exhibited extensive lipid droplet accumulation as demonstrated by Oil Red O staining (Figure 8B, upper panels). In contrast, BIX02189 treatment markedly reduced intracellular lipid accumulation at both D7 and D12. Phase-contrast microscopy revealed that BIX02189-treated cells retained a fibroblast-like morphology and exhibited fewer mature adipocyte characteristics than differentiated control cells (middle panels). Consistent with these observations, DAPI staining demonstrated comparable nuclear distribution and cell density between groups (lower panels), suggesting that the reduction in lipid accumulation was not primarily attributable to cell loss but rather to suppression of adipogenic differentiation. These representative images demonstrate the observed differences among the individual single-treatment groups. Collectively, these findings demonstrate that MEK5 signaling is activated during human adipocyte differentiation and that pharmacological inhibition of MEK5 by BIX02189 effectively suppresses adipogenic differentiation and lipid accumulation in hASCs. These results validate the anti-adipogenic effects of BIX02189 in a human cell model and support a conserved role of MEK5 signaling in adipocyte differentiation across species.

### 2.9. BIX02189 Attenuates Diet-Induced Lipid Accumulation in a Zebrafish Model

**Figure 9:**
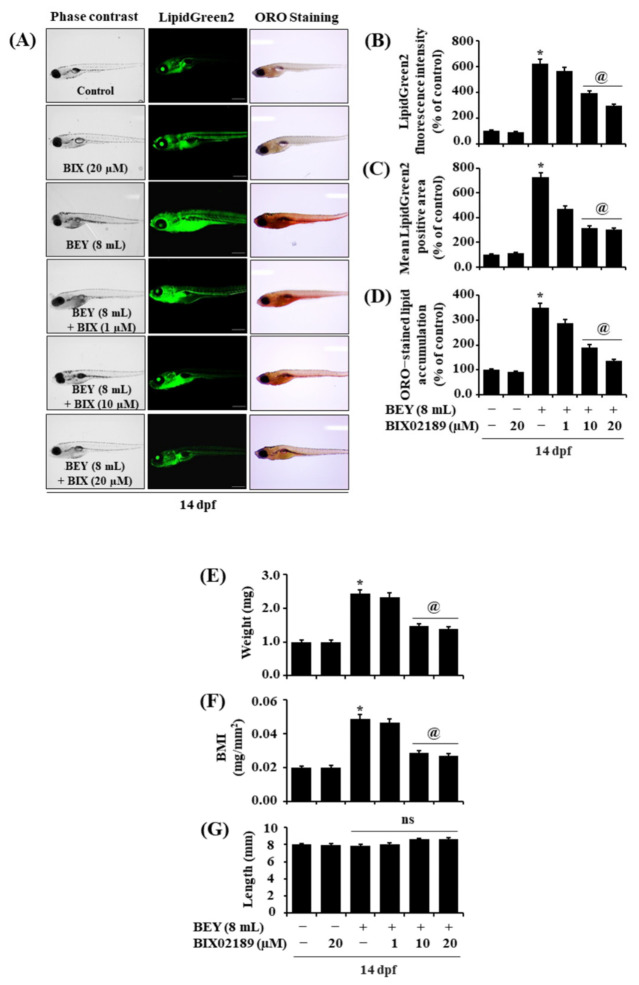
BIX02189 attenuates diet-induced lipid accumulation in a zebrafish obesity model. (**A**) Representative bright-field (**left**), LipidGreen2 fluorescence (**middle**), and Oil Red O-stained (**right**) images of zebrafish larvae at 14 days post-fertilization (14 dpf). A diet-induced obesity model was established by feeding larvae with boiled egg yolk (BEY). Larvae were treated with BIX02189 alone (20 μM) or co-treated with BEY and BIX02189 (1, 10, or 20 μM). (**B**) Quantitative analysis of LipidGreen2 fluorescence intensity. (**C**) Quantitative analysis of the LipidGreen2-positive area. (**D**) Quantitative analysis of Oil Red O-positive lipid accumulation. (**E**) Body weight (mg) of zebrafish larvae at 14 dpf. (**F**) Body mass index (BMI; mg/mm^2^) of zebrafish larvae at 14 dpf. (**G**) Body length (mm) of zebrafish larvae at 14 dpf. BIX02189 reduced BEY-induced lipid accumulation, body weight, and BMI in a concentration-dependent manner without significantly affecting body length. Data are presented as mean ± SE from at least three independent experiments. * *p* < 0.05 versus control; @ *p* < 0.05 versus BEY.

To determine whether the anti-adipogenic effects of BIX02189 observed in vitro could be translated into an in vivo setting, a zebrafish diet-induced obesity model was established using boiled egg yolk (BEY) feeding. Lipid accumulation and obesity-related phenotypes were evaluated at 14 days post-fertilization (14 dpf). As shown in the representative bright-field, LipidGreen2 fluorescence, and Oil Red O staining images (Figure 9A), BEY-fed zebrafish larvae exhibited markedly increased lipid accumulation compared with untreated control larvae. Enhanced LipidGreen2 fluorescence and intense Oil Red O staining were predominantly observed in the abdominal and visceral regions, confirming successful induction of obesity-like lipid deposition. In contrast, treatment with BIX02189 alone (20 μM) did not noticeably alter lipid accumulation relative to control larvae. Importantly, co-treatment with BIX02189 significantly attenuated BEY-induced lipid accumulation in a concentration-dependent manner. While 1 μM BIX02189 produced a modest reduction in lipid deposition, treatment with 10 μM and 20 μM markedly decreased both LipidGreen2 fluorescence and Oil Red O-positive staining (Figure 9A). Quantitative analysis confirmed that BEY feeding significantly increased LipidGreen2 fluorescence intensity (Figure 9B), mean LipidGreen2-positive area (Figure 9C), and Oil Red O-stained lipid accumulation (Figure 9D), whereas BIX02189 significantly reduced all three parameters in a dose-dependent manner. Representative images from three independent zebrafish experiments for Figure 9A are shown in Figure S3. To further assess obesity-related phenotypes, body weight, body mass index (BMI), and body length were measured at 14 dpf. BEY feeding significantly increased body weight (Figure 9E) and BMI (Figure 9F) compared with the control group, indicating successful induction of an obese phenotype. Co-treatment with BIX02189 significantly attenuated BEY-induced increases in both body weight and BMI in a concentration-dependent manner. In contrast, body length showed only minor changes among the experimental groups (Figure 9G), suggesting that the reduction in BMI was primarily attributable to decreased lipid accumulation and body weight rather than impaired larval growth. Collectively, these findings demonstrate that BIX02189 effectively suppresses diet-induced lipid accumulation and obesity-associated phenotypes in vivo while exerting minimal effects on normal developmental growth. These results identify the MEK5 signaling pathway as a novel mediator of adipocyte differentiation and lipid accumulation warranting further investigation.

## 3. Discussion

Obesity is a major global health challenge and is closely associated with insulin resistance, type 2 diabetes mellitus, cardiovascular disease, nonalcoholic fatty liver disease, and several types of cancer. Adipose tissue is now recognized as a highly dynamic endocrine organ that actively regulates systemic metabolism rather than serving merely as an energy storage depot. Consequently, identification of signaling pathways that regulate adipocyte differentiation remains an important strategy for the development of anti-adipogenic therapies [27]. In the present study, we demonstrate that pharmacological inhibition of MEK5 by BIX02189 suppresses adipogenesis and lipid accumulation in murine 3T3-L1 cells, human adipose-derived stem cells (hASCs), and a zebrafish diet-induced obesity model. Mechanistically, these effects were associated with inhibition of STAT3/STAT5 signaling, suppression of adipogenic and lipogenic gene expression, reduction in intracellular ATP levels, activation of AMPK, and downregulation of ACC.

The MAPK family plays critical roles in cellular differentiation and metabolic regulation. Previous studies demonstrated that ERK1/2, JNK, and p38 MAPK pathways are important regulators of cellular differentiation and metabolic responses. While these pathways have been extensively investigated in adipocyte biology, relatively little attention has been directed toward the MEK5–ERK5 pathway [28]. MEK5 signaling is involved in cardiovascular development, skeletal muscle adaptation, stress responses, and tumor progression [29,30]. More recently, it was suggested that MEK5–ERK5 signaling may possess broader metabolic functions than previously appreciated. In the present study, phosphorylation of MEK5 progressively increased during adipocyte differentiation in both 3T3-L1 cells and hASCs, whereas pharmacological inhibition of MEK5 markedly suppressed adipocyte differentiation and lipid accumulation. To the best of our knowledge, our findings suggest that MEK5 signaling plays an important regulatory role in adipogenesis and obesity-associated lipid accumulation.

Adipocyte differentiation is governed by a transcriptional cascade centered on PPAR-γ and C/EBP-α. PPAR-γ is indispensable for adipocyte differentiation, whereas the central role of C/EBP family members is in adipocyte lineage commitment and maturation [31]. Activation of the MEK5 pathway contributes to adipocyte differentiation by regulating the expression of the master adipogenic transcription factors PPAR-γ and C/EBP-α, thereby promoting adipogenic gene expression and lipid deposition [32,33]. Consistent with these findings, differentiation of 3T3-L1 cells was accompanied by progressive induction of PPAR-γ and C/EBP-α. Importantly, BIX02189 significantly reduced both protein and mRNA expression of these transcription factors. Since PPAR-γ and C/EBP-α regulate numerous genes involved in lipid uptake, triglyceride synthesis, and adipocyte maturation, inhibition of these master regulators likely represents a major mechanism underlying the anti-adipogenic effects of BIX02189.

STAT signaling has emerged as an important regulatory pathway in adipocyte biology. Previous studies have demonstrated that STAT proteins are involved in adipocyte differentiation, and subsequent investigations identified STAT5 as a key regulator of adipogenesis [34,35]. More recently, the importance of STAT signaling in adipocyte differentiation, obesity-associated inflammation, and metabolic dysfunction has been highlighted in the development of obesity [36]. In the present study, phosphorylation of both STAT3 and STAT5 progressively increased during adipocyte differentiation and was markedly suppressed by BIX02189 treatment. These findings suggest that MEK5 signaling may positively regulate adipogenesis through activation of STAT3/STAT5 signaling. Although the precise molecular interaction between MEK5 and STAT proteins remains to be elucidated, our data identify a previously unrecognized MEK5–STAT3/STAT5 signaling axis involved in adipocyte differentiation.

In addition to suppressing adipogenic transcription factors, BIX02189 broadly inhibited lipogenic pathways. Expression of FAS, perilipin A, leptin, and ACC was significantly reduced following MEK5 inhibition. FAS functions as a key enzyme responsible for de novo fatty acid synthesis, whereas the critical role of perilipin A is in lipid droplet formation and stabilization [37,38]. Past studies established leptin as a major adipocyte-derived hormone regulating energy balance and adipose tissue homeostasis [39]. Therefore, simultaneous suppression of these molecules indicates that MEK5 inhibition not only impairs adipocyte differentiation but also suppresses lipid synthesis, lipid storage, and adipocyte endocrine activity.

One of the most intriguing findings of the present study is the relationship between MEK5 signaling and cellular energy metabolism. Intracellular ATP levels remained at substantial levels throughout adipocyte differentiation in control cells but were significantly reduced following BIX02189 treatment. Notably, the ATP-lowering effect of BIX02189 was comparable to that induced by 2-deoxy-D-glucose (2-DG), a well-established glycolytic inhibitor and inducer of metabolic stress. Consistent with ATP depletion, BIX02189 markedly increased AMPK phosphorylation.

AMPK is widely recognized as a master regulator of cellular energy homeostasis. The AMPK functions as a metabolic sensor that coordinates cellular responses to energetic stress [40]. More recently, previous analysis emphasized the therapeutic importance of AMPK activation in obesity and metabolic disease [41]. In the present study, activation of AMPK was accompanied by reduced intracellular ATP levels, suggesting that inhibition of MEK5 induces a state of metabolic stress. Unexpectedly, BIX02189 reduced LKB1 phosphorylation while simultaneously increasing AMPK activation. Under classical conditions, LKB1 functions as a major upstream kinase of AMPK. However, studies demonstrated that AMPK can also be activated through alternative pathways involving CaMKKβ, TAK1, mitochondrial dysfunction, oxidative stress, and alterations in AMP/ATP ratios. Therefore, our findings suggest that MEK5 inhibition induces metabolic stress that may activate AMPK, at least in part, through a mechanism independent of canonical LKB1 signaling [42,43]. Although the precise upstream mechanism remains unknown, our findings reveal a previously unrecognized connection between MEK5 signaling and cellular energy sensing pathways. Activation of AMPK has important consequences for lipid metabolism. AMPK acts as a central coordinator of cellular metabolism, whereas the suppression of fatty acid synthesis by AMPK is through regulation of ACC [44,45,46]. Interestingly, BIX02189 not only altered ACC phosphorylation but also significantly reduced ACC protein and mRNA expression. These findings suggest that MEK5 inhibition suppresses lipogenesis through both signaling-dependent and transcriptional mechanisms. Such dual regulation likely contributes substantially to the potent anti-adipogenic effects observed in the present study.

The translational significance of our findings is strengthened by the consistency of the results across multiple biological systems. Similar anti-adipogenic effects were observed in murine 3T3-L1 cells, human adipose-derived stem cells, and zebrafish larvae. As discussed by various authors, zebrafish have emerged as a powerful vertebrate model for obesity research because adipogenesis, lipid metabolism, and energy homeostasis pathways are highly conserved between zebrafish and mammals [47]. In the present study, BIX02189 significantly reduced LipidGreen2 fluorescence intensity, LipidGreen2-positive area, Oil Red O-stained lipid accumulation, body weight, and BMI in BEY-fed zebrafish. Importantly, body length was minimally affected, suggesting that the reduction in adiposity was not attributable to developmental toxicity or growth retardation. These observations support the possibility that MEK5 inhibition selectively suppresses excessive lipid accumulation while preserving normal developmental growth.

Several limitations should be acknowledged. First, although BIX02189 is widely recognized as a selective MEK5 inhibitor, genetic approaches such as MEK5 knockdown, knockout, or overexpression studies were not performed. Second, ERK5, the principal downstream effector of MEK5, was not examined in the present study. Third, the molecular mechanism linking MEK5 inhibition to ATP depletion and AMPK activation remains unclear. Finally, although zebrafish provide valuable in vivo evidence, validation in mammalian obesity models will be necessary before clinical translation can be considered.

In conclusion, the present study identifies MEK5 as a novel regulator of adipocyte differentiation, lipogenesis, and cellular energy metabolism. Based on our findings, we propose a working model in which MEK5 inhibition suppresses STAT3/STAT5-dependent adipogenic signaling while inducing ATP depletion-associated metabolic stress, thereby leading to AMPK activation and subsequent inhibition of ACC-mediated lipogenesis. Although our data suggest that AMPK activation occurs, at least in part, through a mechanism independent of canonical LKB1 signaling, the precise upstream regulatory pathways, including ATP-dependent energy stress signaling, were not directly investigated in the present study. Therefore, the proposed signaling cascade should be considered a mechanistic model supported by the current experimental evidence rather than a fully established molecular mechanism. These coordinated events ultimately suppress adipocyte differentiation and lipid accumulation both in vitro and in vivo. Collectively, our findings expand the biological functions of MEK5 beyond its established roles in growth and stress signaling and suggest that targeting the MEK5 pathway may represent a candidate target for modulating adipogenesis in the context of metabolic disease.

## 4. Materials and Methods

### 4.1. Reagents and Antibodies

BIX02189, a selective MEK5 inhibitor, was purchased from APExBIO (Houston, TX, USA). Dexamethasone, 3-isobutyl-1-methylxanthine (IBMX), insulin, and Oil Red O were obtained from Sigma-Aldrich (St. Louis, MO, USA). Hematoxylin solution was purchased from Abcam (Cambridge, UK). Enhanced chemiluminescence (ECL) detection reagents were obtained from Advansta (San Jose, CA, USA). The primary and secondary antibodies used in this study are listed in Table S1. Antibodies against adipogenic, lipogenic, and signaling proteins, including MEK5, phospho-MEK5, STAT3, phospho-STAT3, STAT5, phospho-STAT5, AMPK, phospho-AMPK, LKB1, phospho-LKB1, ACC, phospho-ACC, PPAR-γ, C/EBP-α, FAS, and perilipin A, were used for immunoblot analysis according to the manufacturers’ instructions.

### 4.2. Cell Culture and Adipocyte Differentiation of 3T3-L1 Preadipocytes and Human Adipose-Derived Stem Cells (hASCs)

Adipogenic differentiation of 3T3-L1 preadipocytes and hASCs was performed as previously described [48], with minor modifications. Mouse 3T3-L1 preadipocytes (ATCC, Manassas, VA, USA) were cultured in Dulbecco’s Modified Eagle Medium (DMEM; Gibco, Rockville, MD, USA) supplemented with 10% fetal bovine serum (FBS; Gibco) and 1% penicillin–streptomycin (P/S; Welgene, Daegu, Republic of Korea) at 37 °C in a humidified atmosphere containing 5% CO_2_. Upon reaching confluence, cells were maintained for an additional 48 h to achieve growth arrest prior to adipogenic induction. To induce adipocyte differentiation, growth-arrested cells were incubated in adipogenic induction medium consisting of DMEM supplemented with 10% FBS, 0.5 mM 3-isobutyl-1-methylxanthine (IBMX), 0.5 μM dexamethasone, and 5 μg/mL insulin (MDI). During the differentiation period, cells were treated with vehicle control (0.1% DMSO) or BIX02189 at the indicated concentrations. After 2 days (D2), the induction medium was replaced with maintenance medium containing DMEM supplemented with 10% FBS and 5 μg/mL insulin in the presence or absence of BIX02189. Thereafter, cells were cultured in DMEM containing 10% FBS, and the medium was replaced every 2 days while maintaining the respective treatments. Fully differentiated adipocytes were harvested on day 8 (D8) for subsequent analyses.

Human adipose-derived stem cells (hASCs) were isolated from abdominal subcutaneous adipose tissue obtained from donors undergoing surgical procedures at Keimyung University Dongsan Hospital (KUDH, Daegu, Republic of Korea). The study protocol was approved by the Institutional Review Board of KUDH (IRB No. 2021-02-063-018), and written informed consent was obtained from all participants before sample collection. Isolated hASCs were cultured in DMEM supplemented with 10% heat-inactivated FBS and 1% P/S under the same culture conditions as described above. For adipogenic differentiation, hASCs were cultured in adipogenic differentiation medium (DM-2; ZenBio, Durham, NC, USA) for 7 days (D0–D7). The medium was then replaced with adipocyte maintenance medium (AM-1; ZenBio), and cells were further cultured for an additional 5 days (D7–D12) in the presence or absence of BIX02189 at the indicated concentrations. The culture medium was refreshed according to the manufacturer’s instructions. After 12 days of differentiation, mature adipocytes were collected and subjected to morphological, biochemical, and molecular analyses.

### 4.3. Evaluation of Lipid Accumulation by Oil Red O Staining

Intracellular lipid accumulation in differentiated 3T3-L1 adipocytes and human adipose-derived stem cell (hASC)-derived adipocytes was evaluated by Oil Red O staining. Briefly, cells were washed twice with phosphate-buffered saline (PBS) and fixed with 10% neutral-buffered formaldehyde for 2 h at room temperature. After fixation, cells were rinsed with 60% isopropanol, air-dried, and incubated with freshly prepared Oil Red O working solution (Sigma-Aldrich, St. Louis, MO, USA) for 1 h at room temperature. Following staining, the cells were washed thoroughly with distilled water to remove excess dye. Lipid droplet accumulation was visualized and photographed using a phase-contrast microscope (Nikon, Tokyo, Japan). To visualize cell nuclei, the stained cells were counterstained with hematoxylin solution (Abcam, Cambridge, UK) for 5 min and subsequently washed several times with distilled water. Representative images were captured under identical microscopic settings, including magnification, exposure time, and image acquisition parameters. For quantitative analysis, Oil Red O-positive staining intensity and lipid-positive area were measured using ImageJ software (v.1.6.0.24; National Institutes of Health, Bethesda, MD, USA) and expressed as a percentage of the control group.

### 4.4. Cell Viability Assay

Cell viability was determined using the trypan blue exclusion assay. Briefly, 3T3-L1 preadipocytes and hASCs were seeded in 24-well plates and cultured under the differentiation conditions described above in the presence or absence of BIX02189. At the end of the differentiation period (day 8 for 3T3-L1 cells and day 12 for hASCs), cells were harvested by trypsinization and mixed with 0.4% trypan blue solution (Gibco, Grand Island, NY, USA). The stained cell suspensions were loaded onto a hemocytometer, and viable (unstained) and non-viable (blue-stained) cells were counted under a light microscope (Nikon, Tokyo, Japan). The viability of each treatment group was expressed as a percentage relative to the vehicle-treated control group. All experiments were performed independently at least three times.

### 4.5. Measurement of Intracellular Triglyceride Content

Intracellular triglyceride (TG) content was quantified using an AdipoRed Assay Reagent kit (Lonza, Basel, Switzerland) according to the manufacturer’s instructions. A panel of 19 protein kinase inhibitors, including BIX02189, was initially screened for anti-adipogenic activity during 3T3-L1 adipocyte differentiation. Differentiated cells treated with vehicle or individual inhibitors were incubated with AdipoRed reagent (Lonza, Basel, Switzerland) for 10 min at room temperature, and intracellular triglyceride (TG) accumulation was quantified by measuring fluorescence using a Victor3 multilabel plate reader (PerkinElmer, Waltham, MA, USA) at excitation and emission wavelengths of 485 and 572 nm, respectively. Based on the primary screening results, BIX02189 was selected for further investigation (Figure 1B). To validate its anti-adipogenic activity, 3T3-L1 cells were differentiated in the presence of increasing concentrations of BIX02189, and intracellular TG accumulation was assessed using the same assay (Figure 2B). Fluorescence values were normalized to the vehicle-treated control group and expressed as percentages of the control. All experiments were performed independently at least three times, and the results are presented as relative intracellular TG content. Data are presented as the mean ± standard error (SE) from three independent experiments.

### 4.6. Cell Lysis and Protein Extraction

At the indicated time points, 3T3-L1 cells or hASCs were washed twice with phosphate-buffered saline (PBS) and lysed using modified RIPA buffer (50 mM Tris-HCl, pH 7.4, 150 mM NaCl, 0.1% sodium dodecyl sulfate, 0.25% sodium deoxycholate, 1% Triton X-100, 1% Nonidet P-40, 1 mM EDTA, 1 mM EGTA, and protease inhibitor cocktail). The cell lysates were incubated on ice for 30 min with intermittent vortexing and subsequently centrifuged at 12,000× *g* for 20 min at 4 °C. The resulting supernatants were collected as whole-cell protein extracts and stored at −80 °C until use. Protein concentrations were determined using the Bradford protein assay kit (Bio-Rad Laboratories, Hercules, CA, USA) according to the manufacturer’s instructions. Bovine serum albumin (BSA) was used as the protein standard.

### 4.7. Western Blot Analysis

Equal amounts of protein (35 μg) were separated by 10% sodium dodecyl sulfate–polyacrylamide gel electrophoresis (SDS–PAGE) and transferred onto nitrocellulose membranes (MilliporeSigma, Burlington, MA, USA). Following transfer, the membranes were washed with Tris-buffered saline containing 0.05% Tween-20 (TBST) and blocked with 5% non-fat skim milk prepared in TBST for 1 h at room temperature. The membranes were then incubated overnight at 4 °C with the appropriate primary antibodies listed in Table S1. After washing three times with TBST, the membranes were incubated with horseradish peroxidase (HRP)-conjugated secondary antibodies for 2 h at room temperature. Following additional washes with TBST, immunoreactive protein bands were detected using enhanced chemiluminescence (ECL) reagents (Advansta, San Jose, CA, USA) according to the manufacturer’s instructions. Chemiluminescent signals were visualized using an imaging system, and band intensities were quantified using ImageJ software (v.1.6.0.24; National Institutes of Health, Bethesda, MD, USA). Protein expression levels were normalized to β-actin, which served as the internal loading control. For phosphorylated proteins, the levels of phospho-proteins were normalized to their corresponding total protein levels prior to normalization against β-actin where appropriate. All Western blot experiments were performed independently at least three times, and representative blots are shown in the figures.

### 4.8. Reverse Transcription–Polymerase Chain Reaction (RT-PCR) Analysis

Total RNA was isolated from 3T3-L1 cells using RNAiso Plus reagent (TaKaRa Bio Inc., Kusatsu, Shiga, Japan) according to the manufacturer’s instructions. Briefly, 3 μg of total RNA was reverse-transcribed into complementary DNA (cDNA) using random hexamer primers and reverse transcriptase. The resulting cDNA was amplified by PCR using gene-specific primers listed in Table S2. PCR products were separated on 1.5% agarose gels and visualized under UV illumination. Band intensities were quantified using ImageJ software (v.1.6.0.24; National Institutes of Health, Bethesda, MD, USA) and normalized to β-actin, which served as the internal control.

### 4.9. Luminescence-Based ATP Assay

3T3-L1 cells were seeded in 96-well plates and induced to differentiate in the presence or absence of BIX02189. Intracellular ATP levels were measured on days 2, 5, and 8 using a luminescence-based ATP assay kit (ATPLite™ 1step; PerkinElmer, Waltham, MA, USA) according to the manufacturer’s instructions. Briefly, the ATP detection reagent was added directly to the cells, followed by incubation for 2 min at room temperature. Luminescence was measured using a Victor3 multilabel plate reader (PerkinElmer, Waltham, MA, USA). ATP levels were normalized to the vehicle-treated control group and expressed as a percentage of control.

### 4.10. Zebrafish Maintenance and Embryo Collection

Wild-type zebrafish (*Danio rerio*; AB strain) were obtained from Chungnam National University (CNU) and maintained in a recirculating aquatic system at 28.5 °C under a 14 h light/10 h dark photoperiod and fed twice daily. For embryo collection, adult male and female zebrafish were paired at a ratio of 1:1 in breeding tanks overnight and allowed to spawn the following morning. Fertilized embryos were collected, rinsed with embryo medium (E3 medium), and maintained in E3 medium (60 embryos per dish) at 28.5 °C until use. All animal experiments were conducted in accordance with institutional guidelines and were approved by the Institutional Animal Care and Use Committee (IACUC) of Keimyung University (Approval No. KM2025-016).

### 4.11. LipidGreen2 Staining and Quantification of Lipid Accumulation

Lipid accumulation in zebrafish larvae was assessed using LipidGreen2 staining. Briefly, larvae were incubated with 1 μM LipidGreen2 (Biomax, Seoul, Republic of Korea) prepared in E3 medium and protected from light during staining. After staining, the larvae were washed three times with fresh E3 medium for 10 min each to remove excess dye. The larvae were subsequently anesthetized on ice and mounted in 3% methylcellulose for imaging. Fluorescence images were captured using an inverted fluorescence microscope under identical imaging conditions. Lipid accumulation was quantified by measuring both LipidGreen2 fluorescence intensity and lipid-positive area using ImageJ software (v.1.6.0.24; National Institutes of Health, Bethesda, MD, USA). The values obtained from each treatment group were normalized to those of the control group and expressed as percentages of control. All experiments were performed independently at least three times.

### 4.12. Oil Red O Staining of Zebrafish Larvae

Lipid accumulation in zebrafish larvae was evaluated by Oil Red O (ORO) staining. Briefly, larvae were fixed in 10% formaldehyde, dehydrated in 60% isopropanol, and stained with Oil Red O solution (Sigma-Aldrich, St. Louis, MO, USA) for 2 h. After washing with distilled water, images were captured under a stereomicroscope (Nikon, Tokyo, Japan). Lipid accumulation was quantified by measuring the ORO-positive area using ImageJ software (v.1.6.0.24; National Institutes of Health, Bethesda, MD, USA) and expressed as a percentage of the control group.

### 4.13. Statistical Analysis

All experiments were performed independently at least three times. Data are presented as the mean ± standard error (SE). Statistical analyses were performed using SPSS software (version 11.5; SPSS Inc., Armonk, NY, USA). Differences among multiple groups were analyzed by one-way analysis of variance (ANOVA) followed by Dunnett’s post hoc test. A *p*-value < 0.05 was considered statistically significant.

## Data Availability

The original contributions presented in this study are included in the article/Supplementary Material. Further inquiries can be directed to the corresponding authors.

## Supplementary Materials

The following supporting information can be downloaded at: https://www.mdpi.com/article/10.3390/ijms27146468/s1.
