# Supplementary material for: BIX02189 Suppresses Adipogenesis and Lipid Accumulation Through Inhibition of MEK5-STAT3/STAT5 Signaling and Activation of AMPK in Adipocytes and Zebrafish
Source: Int J Mol Sci. 2026 Jul 21;27(14):6468. doi: 10.3390/ijms27146468 (PMC13409913; doi:10.3390/ijms27146468)
Supplement: Supplementary file 1 [file ijms-27-06468-s001.zip › Supplementry Figures.pptx]

## Slide 1
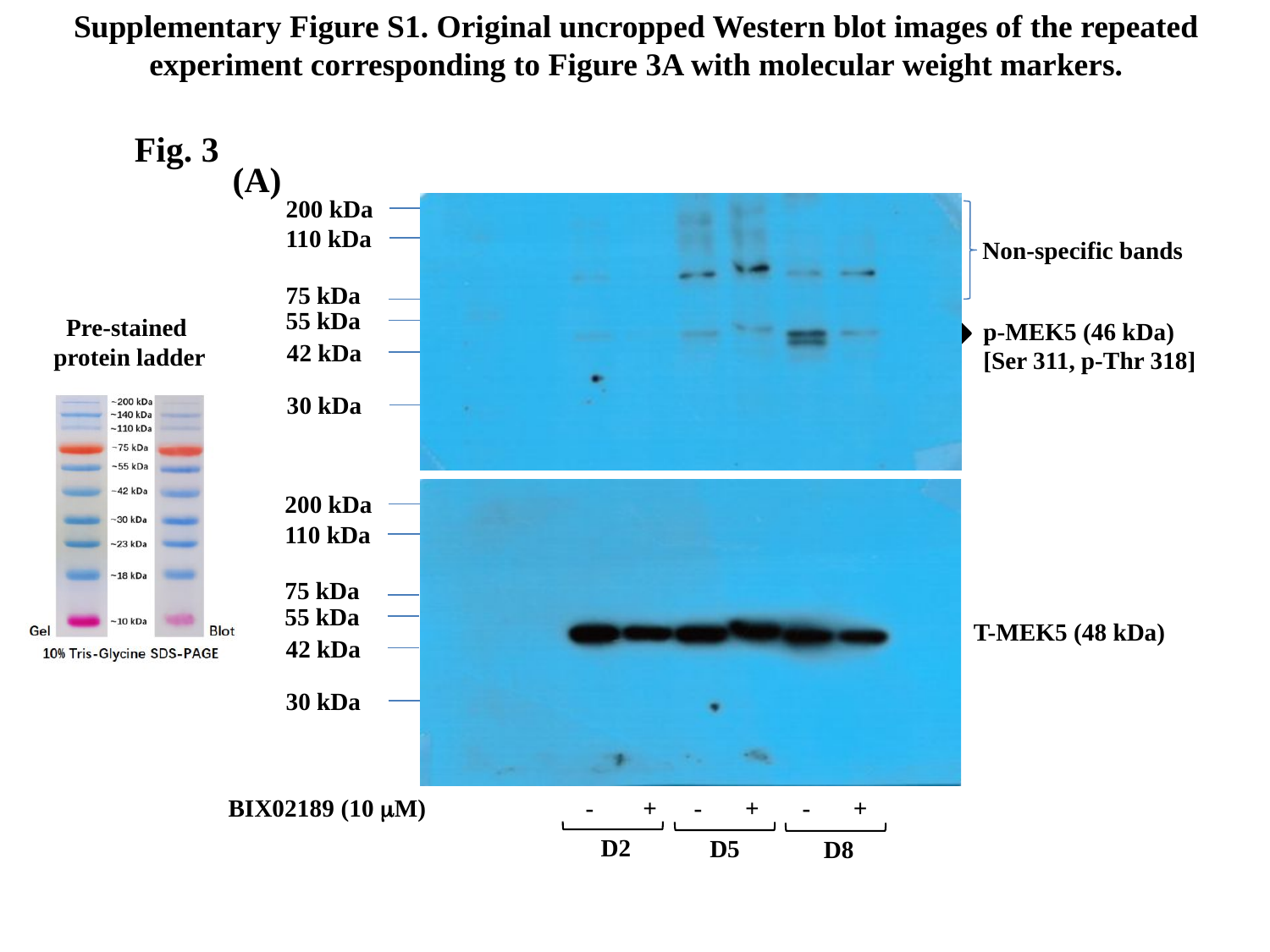

Supplementary Figure S1. Original uncropped Western blot images of the repeated experiment corresponding to Figure 3A with molecular weight markers.
Fig. 3
(A)
200 kDa
110 kDa
Non-specific bands
75 kDa
55 kDa
Pre-stained
protein ladder
p-MEK5 (46 kDa)
[Ser 311, p-Thr 318]
42 kDa
30 kDa
200 kDa
110 kDa
75 kDa
55 kDa
T-MEK5 (48 kDa)
42 kDa
30 kDa
BIX02189 (10 μM) - + - + - +
 D2
D5
 D8

## Slide 2
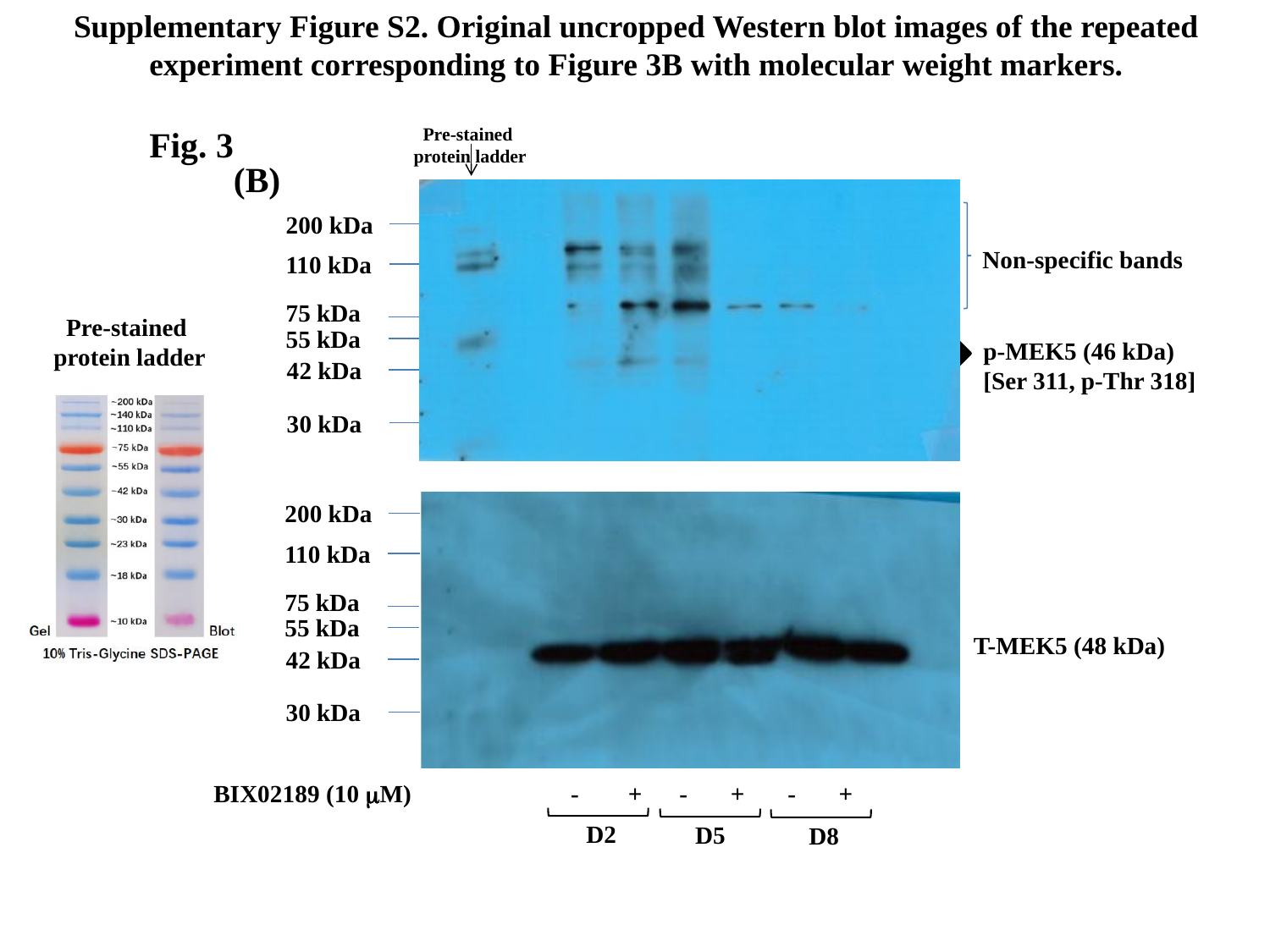

Supplementary Figure S2. Original uncropped Western blot images of the repeated experiment corresponding to Figure 3B with molecular weight markers.
Fig. 3
Pre-stained
protein ladder
(B)
200 kDa
Non-specific bands
110 kDa
75 kDa
Pre-stained
protein ladder
55 kDa
p-MEK5 (46 kDa)
[Ser 311, p-Thr 318]
42 kDa
30 kDa
200 kDa
110 kDa
75 kDa
55 kDa
T-MEK5 (48 kDa)
42 kDa
30 kDa
BIX02189 (10 μM) - + - + - +
 D2
D5
 D8

## Slide 3
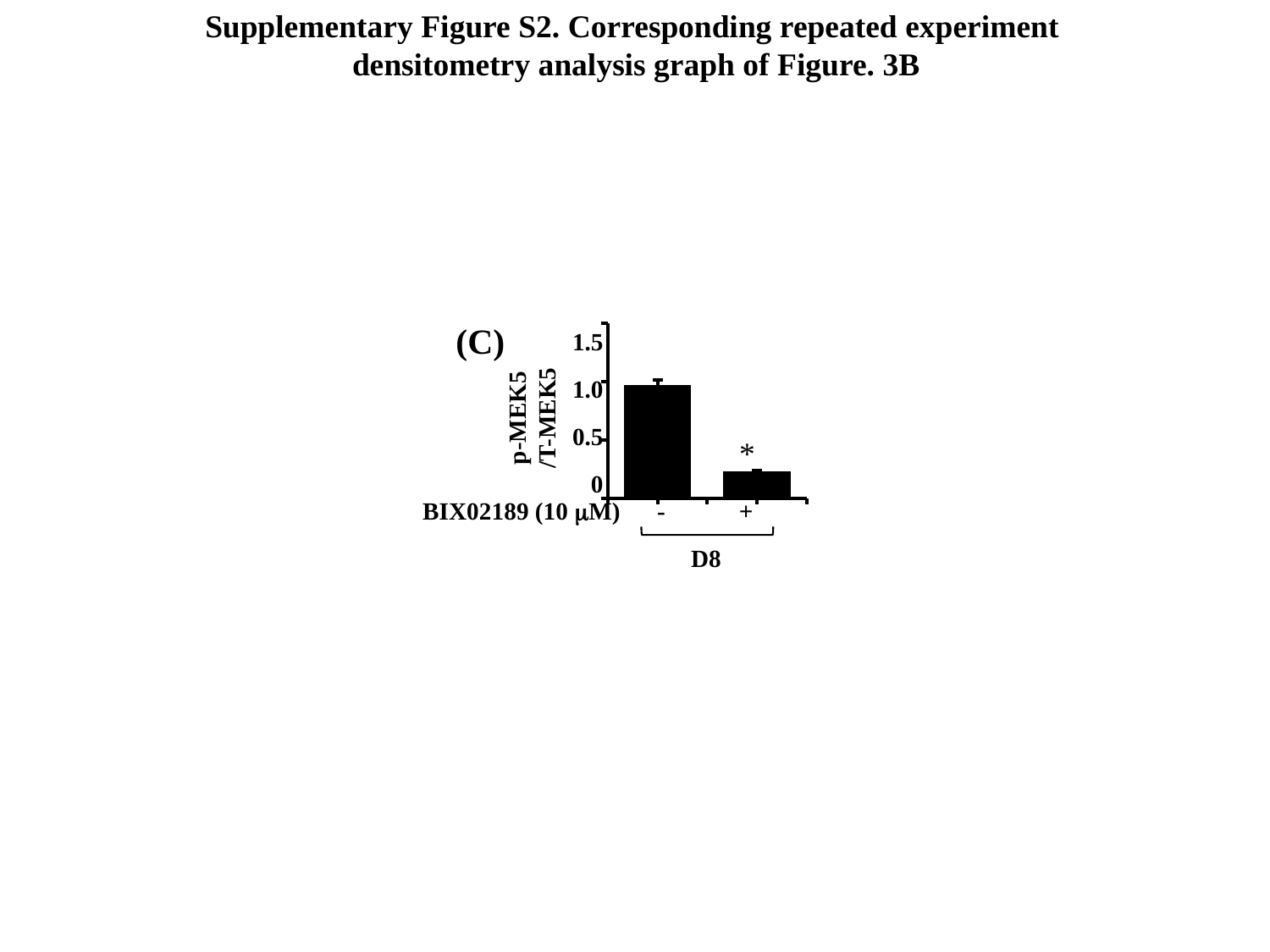

Supplementary Figure S2. Corresponding repeated experiment
densitometry analysis graph of Figure. 3B
(C)
1.5
1.0
0.5
0
### Chart
| Category | |
|---|---|p-MEK5
/T-MEK5
*
BIX02189 (10 μM) - +
D8

## Slide 4
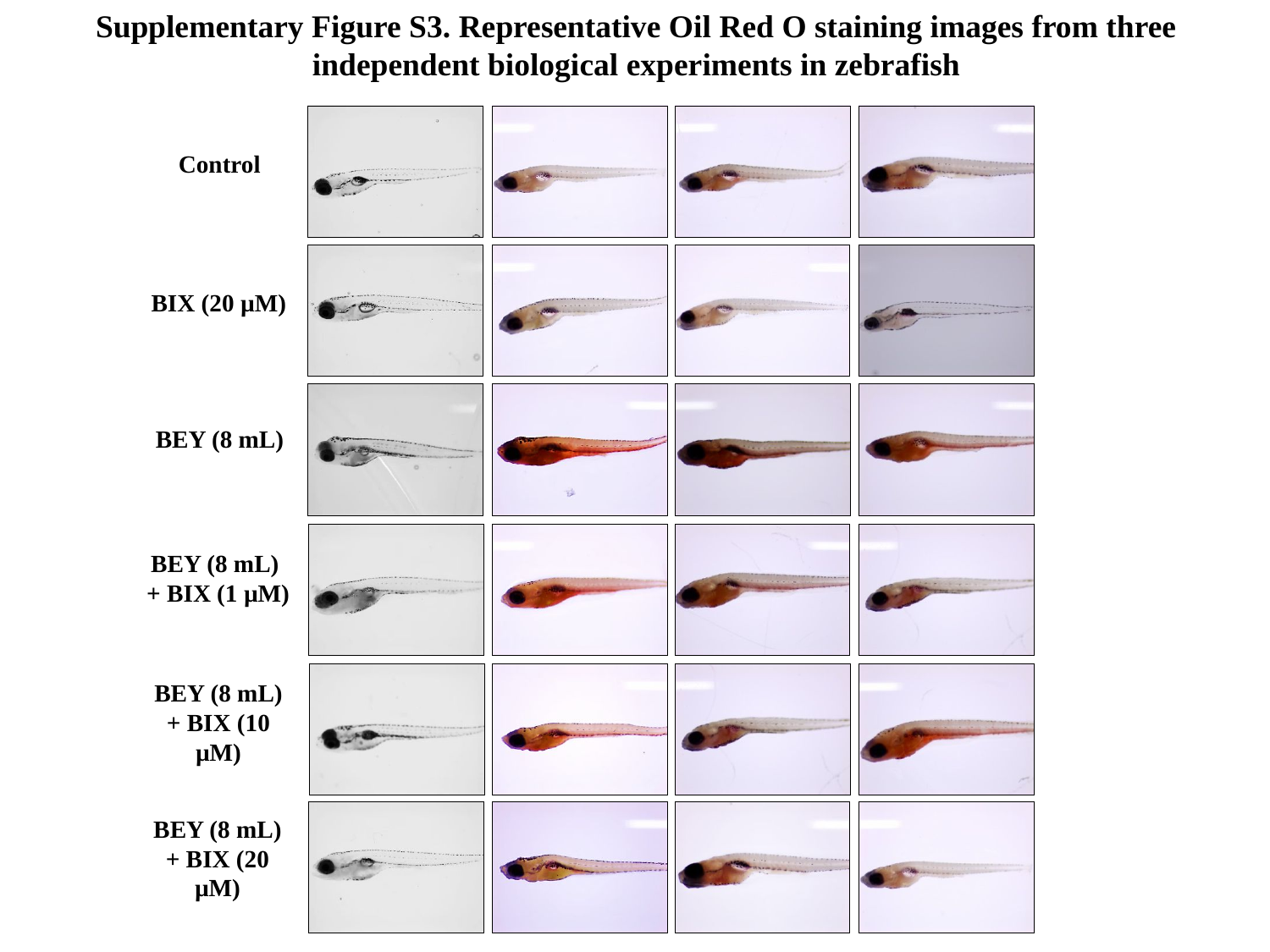

Supplementary Figure S3. Representative Oil Red O staining images from three independent biological experiments in zebrafish
Control
BIX (20 µM)
BEY (8 mL)
BEY (8 mL)
+ BIX (1 µM)
BEY (8 mL)
+ BIX (10 µM)
BEY (8 mL)
+ BIX (20 µM)
