# Supplementary material for: BIX02189 Suppresses Adipogenesis and Lipid Accumulation Through Inhibition of MEK5-STAT3/STAT5 Signaling and Activation of AMPK in Adipocytes and Zebrafish
Source: Int J Mol Sci. 2026 Jul 21;27(14):6468. doi: 10.3390/ijms27146468 (PMC13409913; doi:10.3390/ijms27146468)
Supplement: Supplementary file 1 [file ijms-27-06468-s001.zip › Table S1.pdf]

Table S1. Western blot analysis antibodies list

Antibodies	Dilution	Company name	Cat. No.
p-MEK5	1:2,000	Novus bio	Cs321g
T-MEK5	1:2,000	Gene Tex	GTX50752
C/EBP-α	1:2,000	Santa Cruz Biotechnology	sc-61
PPAR-γ	1:2,000	Santa Cruz Biotechnology	sc-7272
p-STAT-3	1:2,000	Santa Cruz Biotechnology	sc-8059
T-STAT-3	1:2,000	Santa Cruz Biotechnology	sc-8019
p-STAT-5	1:2,000	Santa Cruz Biotechnology	sc-81524
T-STAT-5	1:2,000	Santa Cruz Biotechnology	sc-1656
FAS	1:2,000	BD Biosciences	#610962
Perilipin A	1:2,000	Biovision	#3948-200
p-LKB1	1:2,000	Cell Signaling Technology	#3482
T-LKB1	1:2,000	Cell Signaling Technology	#3047
p-AMPK (T172)	1:2,000	Cell signaling	#2535
T-AMPK	1:2,000	Cell signaling	#2793
p-ACC (S79)	1:2,000	Cell signaling	#3661
T-ACC	1:2,000	Cell signaling	#3662
β-actin	1:10,000	Sigma	A5441
Goat anti-rabbit IgG-HRP	1:2,000	Jackson ImmunoResearch Laboratories	111-035-045
Goat anti-mouse IgG-HRP	1:2,000	Jackson ImmunoResearch Laboratories	115-035-062
