# Supplementary material for: BIX02189 Suppresses Adipogenesis and Lipid Accumulation Through Inhibition of MEK5-STAT3/STAT5 Signaling and Activation of AMPK in Adipocytes and Zebrafish
Source: Int J Mol Sci. 2026 Jul 21;27(14):6468. doi: 10.3390/ijms27146468 (PMC13409913; doi:10.3390/ijms27146468)
Supplement: Supplementary file 1 [file ijms-27-06468-s001.zip › Table S2.pdf]

Table S2. RT-PCR primers list

Gene	Sense [5′-3′]	Anti-sense [5′-3′]
C/EBP-α	TTACAACAGGCCAGGTTTCC	CTCTGGGATGGATCGATTGT
PPAR-γ	GGTGAAACTCTGGGAGATTC	CAACCATTGGGTCAGCTCTC
FAS	TTGCTGGCACTACAGAATGC	AACAGCCTCAGAGCGACAAT
Perilipin A	TTCTCGACACACCATGGAAACC	CACGTTATCCGTAACACCCTTCA
Adiponectin	GGAGATGCAGGTCTTCTTGGT	TCCTGATACTGGTCGTAGGTGAA
Leptin	CCAAAACCCTCATCAAGACC	CTCAAAGCCACCACCTCTGT
Actin	TCATGAAGTGTGACGTTGACATCCGT	CCTAGAAGCATTTGCGGTGCACGATG
